# Valuable New Books

**Published:** 1877-06

**Authors:** 


### Editorial.

### Valuable New Books.

It will be observed that we have noticed as *Received* several valuable standard publications which we have not yet noticed in *Review.* This has occurred from the necessities of the case, our space not allowing of extended review of several new and valuable books. We omit comment upon all subjects of general outside professional interest in order to make amends so far as possible to our readers as well as medical book publishers, to whom we and the whole profession, owe so much. We hope to be able in future to give a correct and comprehensive *resumé* of all medical works published in this country or any other as soon as consistent after receiving them, and hope our readers will accept this as apology for past neglect. Those who know the situation best, will forgive us most freely.

